# Transcriptomic responses of the basidiomycete yeast *Sporobolomyces* sp. to the mycotoxin patulin

**DOI:** 10.1186/s12864-016-2550-4

**Published:** 2016-03-09

**Authors:** Giuseppe Ianiri, Alexander Idnurm, Raffaello Castoria

**Affiliations:** Dipartimento di Agricoltura, Ambiente e Alimenti, Università degli Studi del Molise, Via F. De Sanctis snc, 86100 Campobasso, Italy; Division of Cell Biology and Biophysics, School of Biological Sciences, University of Missouri-Kansas City, Kansas City, MO 64110 USA; School of BioSciences, University of Melbourne, Melbourne, VIC 3010 Australia; Present address: Department of Molecular Genetics and Microbiology, Duke University Medical Center, Durham, NC 27710 USA

**Keywords:** Pucciniomycotina, Transcriptome, Mycotoxins, Biocontrol, Patulin

## Abstract

**Background:**

Patulin is a mycotoxin produced by *Penicillium expansum,* the causal agent of blue mold of stored pome fruits, and several other species of filamentous fungi. This mycotoxin has genotoxic, teratogenic and immunotoxic effects in mammals, and its presence in pome fruits and derived products represents a serious health hazard. Biocontrol agents in the Pucciniomycotina, such as the yeasts *Sporobolomyces* sp. strain IAM 13481 and *Rhodosporidium kratochvilovae* strain LS11, are able to resist patulin and degrade it into the less toxic compounds desoxypatulinic acid and ascladiol.

**Results:**

In this investigation we applied a transcriptomic approach based on RNAseq to annotate the genome of *Sporobolomyces* sp. IAM 13481 and then study the changes of gene expression in *Sporobolomyces* sp. exposed to patulin. Patulin treatment leads to ROS production and oxidative stress that result in the activation of stress response mechanisms controlled by transcription factors. Upregulated *Sporobolomyces* genes were those involved in oxidation-reduction and transport processes, suggesting the activation of defense mechanisms to resist patulin toxicity and expel the mycotoxin out of the cells. Other upregulated genes encoded proteins involved in metabolic processes such as those of the glutathione and thioredoxin systems, which are essential to restore the cellular redox homeostasis. Conversely, patulin treatment decreased the expression of genes involved in the processes of protein synthesis and modification, such as transcription, RNA processing, translation, protein phosphorylation and biosynthesis of amino acids. Also, genes encoding proteins involved in transport of ions, cell division and cell cycle were downregulated. This indicates a reduction of metabolic activity, probably due to the high energy requirement by the cells or metabolic arrest while recovering from the insult caused by patulin toxicity.

**Conclusions:**

Complex mechanisms are activated in a biocontrol yeast in response to patulin. The genes identified in this study can pave the way to develop i) a biodetoxification process of patulin in juices and ii) a biosensor for the rapid and cost-effective detection of this mycotoxin.

**Electronic supplementary material:**

The online version of this article (doi:10.1186/s12864-016-2550-4) contains supplementary material, which is available to authorized users.

## Background

Mycotoxins are food contaminants with harmful impact on human and animal health. Their occurrence in commercialized food and feed is a consequence of fungal attacks on crops in the field and/or on stored products. Patulin (PAT) is a mycotoxin produced by species in the fungal genera *Aspergillus* and *Penicillium. P. expansum*, the causative agent of the blue mold disease of stored apples, is the main PAT producer and, as a consequence of its attack, PAT is found in different fruits especially pome fruits and derived products. PAT contamination poses a major risk for children, who consume great quantities of fruit juices.

The mechanisms of PAT toxicity and its effects on living cells have been explored for decades [1, 2]. The main targets of patulin are cellular nucleophiles [3]. In eukaryotic cells, patulin induces oxidative stress by lowering the concentration of the antioxidant peptide glutathione, to which it binds due to its electrophilic reactivity, and through generation of reactive oxygen species (ROS) [4, 5]. ROS generation plays a role in the molecular events leading to apoptotic processes particularly by inducing peroxidation of membrane lipids and oxidative DNA damage [6–8].

Genome-wide analysis of the model yeast *Saccharomyces cerevisiae* on cells exposed to PAT revealed increased levels of transcripts of genes involved in proteasome activity, metabolism of sulfur amino acids, and stress responses, which included transporters involved in detoxification and multidrug resistance, oxidative stress scavengers (thioredoxin and glutathione) and DNA repair genes [9]. Another study in the fission yeast *Schizosaccharomyces pombe* confirmed the ROS-induced PAT toxicity through glutathione (GSH) depletion, and the activation of antioxidants and redox systems controlled by the transcription factor Pap1 [10], which is the ortholog of Yap1, a key transcription factor that mediates oxidative stress responses in *S. cerevisiae* [11, 12] and *Cryptococcus neoformans* [13]. Moreover, in *S. pombe* PAT also causes plasma membrane fluidization [14] and changes in chromatin structure [15].

In mammals, the primary target organs of PAT toxicity are the gastrointestinal tract, kidney, liver and the immune system, with acute symptoms of intestinal hemorrhage, ulceration, nausea, edema, dyspnea, convulsion, and agitation. The carcinogenic risk of PAT is classified in group 3 by the International Agency for Research on Cancer [16], since the evidence for its carcinogenicity is considered inadequate in humans and experimental animals. Long-term consequences of exposure to toxic PAT concentrations may include mutagenicity, genotoxicity, embryotoxicity, and exposure to high dosages may include immunosuppression, immunotoxicity and neurotoxicity [1, 2, 17]. The Joint Food and Agriculture Organization/World Health Organization Expert Committee on Food Additives established a maximum tolerable daily intake for PAT of 0.4 mg/kg body weight/day [18]. Based upon its toxicity profile, legislative regulations in Europe and USA set the highest tolerable levels of PAT in fruit-based products and juices at 50 μg/kg, and for baby food at 10 μg/kg (EC Regulation 1881/2006). Despite this restrictive legislation, recent surveys in Europe and USA revealed that PAT contamination is still a common issue [19–21].

In addition to adverse effects on mammals, PAT inhibits microbial growth, having antibacterial, antiviral and antifungal activities, and damages plant cells [2]. As with other secondary metabolites produced by filamentous fungi, the role of PAT is likely to help in competition against other microbes that those fungi encounter in their environment. A gene cluster encoding proteins for PAT biosynthesis has been identified [2, 22], and mutation of these genes allowed the study of the role of PAT in the pathogenicity of *P. expansum,* which is still controversial and likely dependent on host factors [23–26].

Postharvest control of *P. expansum* infections is crucial to prevent PAT accumulation in stored fruits. Currently, this is achieved through a combination of cold storage and treatment with chemical fungicides. However, because of ethical, technical and health issues, there is an increasing demand for alternative methods to reduce the use of chemicals; the use of biocontrol agents and/or controlled atmosphere are very promising strategies [21].

**Fig. 1:**
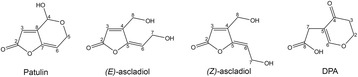
Chemical structures of patulin and of the three breakdown products *(E)*-ascladiol, *(Z)*-ascladiol and desoxypatulinic acid (DPA)

Besides reduction of *P. expansum* attack on stored fruits, strategies to detoxify PAT also could provide safer juices for consumption. A number of microbes have been identified that are naturally resistant to PAT, and some have the ability of degrading it. The influence of biocontrol agents (BCAs) that are effective against *P. expansum* on PAT accumulation is an emerging and attractive field of study, and major findings have been recently reviewed [27]. Two ascomycete yeast species, *Pichia ohmeri* and *Candida sake*, and a bacterium, *Pantoea agglomerans*, were able to reduce PAT accumulation; however, the observed reductions were not attributed to direct PAT metabolization but to the protection of fruits from infection by PAT-producing *P. expansum*, or by PAT absorption by cell wall and/or into the cells [21, 27]*.* Conversely, the BCA *Rhodosporidium kratochvilovae* strain LS11, which is a basidiomycete yeast in subphylum Pucciniomycotina, was able to resist and detoxify PAT *in vitro* through two independent pathways that led to the formation of breakdown products that are much less toxic than the mycotoxin. The isomers *(E)* and *(Z)* of ascladiol, intermediate in PAT biosynthesis, were found as transient products, while desoxypatulinic acid (DPA) was identified as the final product of PAT degradation [28, 29]. Another Pucciniomycotina yeast, *Sporobolomyces* sp. strain IAM 13481, also converts PAT to DPA and ascladiols, but in this case *(E)* ascladiol was found as a transient metabolite, while DPA and *(Z)* ascladiol were the two final breakdown products [30]. More recently, *Rhodosporidium paludigenum* and other Pucciniomycotina species were found to be able to degrade PAT to DPA and/or ascladiols ([31]; our unpublished data). Therefore, it seems likely that different mechanisms, depending on the species under examination, regulate the expression of the two degradation pathways present in Pucciniomycotina yeasts. The hypothesis of the two distinct pathways is corroborated by the chemical structures of DPA and ascladiols, as they derive from the breakage of bonds that are located in separate ends of the PAT molecule (Fig. 1). Interestingly, while the production of DPA was found only in Pucciniomycotina species, which suggests a peculiar feature of this lineage of basidiomycete fungi, ascladiols were detected after incubation of PAT with the ascomycete *Saccharomyces cerevisiae* and with the bacteria *Gluconobacter oxydans* and *Lactobacillus plantarum* [32–34]*,* indicating that the ascladiol-forming degradation pathway is likely to be conserved across fungi and bacteria.

We recently explored the molecular mechanisms involved in PAT degradation by Pucciniomycotina yeasts through a random insertional mutagenesis approach: *Sporobolomyces* sp. IAM 13481 was used as the model organism because of the availability of its genome sequence. However, only a few genes involved in resistance to PAT toxicity were identified [30], suggesting that PAT treatment activates multiple cellular responses that are unlikely to be elucidated in their entirety using a forward genetics strategy.

Therefore, in the present study we analyzed the effects of PAT treatment on *Sporobolomyces* sp. IAM 13481 cells through a genome-wide approach based on the RNA sequencing (RNAseq). Amongst the fungi there are only two studies that investigated the effect of PAT at the genome-wide level, both on *S. cerevisiae*, with one analyzing the highly expressed genes, and the second focusing on the differential transcriptional response of a *sod1*∆ mutant in comparison with the wild type strain and on the scavenging effect of ascorbic acid [9, 35]. The present study represents the first application of the high-throughput sequencing technique RNAseq for a non-conventional yeast that is of interest for food safety. The aims were to provide a comprehensive analysis of the genes, the cellular processes and the cellular compartments that play a crucial role in response to PAT treatment, and to identify potential mechanisms and enzymes that are involved in PAT degradation.

## Results

### Generation of a reference transcriptome

The genome sequence of the monokaryon strain IAM 13481 of *Sporobolomyces* sp. is available on the JGI website at the US Department of Energy (http://genome.jgi-psf.org/Sporo1/Sporo1.home.html). Its size is 21.2 Mb and includes 76 scaffolds, four of which contained about half of the genome, and 5536 gene models predicted and with putative functions annotated using the JGI annotation pipeline. Although a key taxon used for comparative genomics with the rust fungi [36], the genome is still a draft not available in GenBank, and for an accurate transcriptome analysis a gene annotation upgrade was necessary.

Several approaches were performed for the generation of a reference transcriptome, such as the use of both strand-specific and non-strand specific sequencing, combined or not with the existing gene annotation. According to BLAST similarity analyses of resulting assembled transcripts, the most accurate transcriptome was obtained by merging new genomic traits revealed by strand-specific sequencing to the existing *Sporobolomyces* sp. annotation.

Strand-specific libraries were prepared from RNA isolated from *Sporobolomyces* sp. 1) yeast cells grown in the presence and in the absence of 5 μg/ml of PAT (two biological replicates for each of two samples), 2) yeast cells collected from YPD agar medium after 2 days of incubation, 3) ballistospore cells fired on a YPD agar mirror plate, a spore dispersal system that we previously described [37]. The table in Additional file 1 summarizes the samples used for RNA extraction, the type of libraries generated and their use for downstream analysis (i.e. genome annotation and/or differential gene expression analysis). The transcriptome datasets are available at the NCBI Sequence Read Archive (SRA), under accession number SRP061533.

Gene annotation was performed using the Tuxedo tools. In particular, reads were cleaned and then mapped to the *Sporobolomyces* genome sequence using Tophat [38]. Approximately 120 million reads (~20 million per sample) were mapped to single locations in the *Sporobolomyces* sp. IAM 13481 genome, for a > 95.0 % overall read mapping rate. Tophat outputs were used as inputs for Cufflinks to generate a primary transcriptome for each growth condition; in order to use the existing annotation as a guide for the assembling and for discovering novel transcripts, the RABT (Reference Annotation Based Transcript) assembly was performed. All the Cufflinks outputs were merged in a single final transcriptome through Cuffmerge. The new generated transcriptome consisted of 6595 unique genes and a total of 14462 gene models due to alternative splicing or variable 5′ and 3′ untranslated regions (UTRs), whose sequences are found in the Additional file 2.

### Transcriptome analysis of *Sporobolomyces* sp. IAM 13481 incubated in the presence of the mycotoxin PAT

**Fig. 2:**
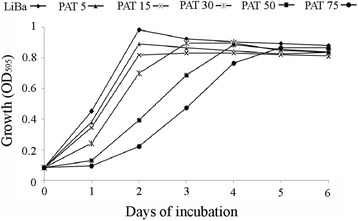
Growth (OD_595_) of *Sporobolomyces* sp. after six days of incubation in LiBa medium in the presence of 0, 5, 15, 30, 50 and 75 μg/ml of PAT

As a first step the effects of PAT on *Sporobolomyces* sp. IAM 13481 were evaluated by monitoring the yeast growth in the presence of different concentrations of the mycotoxin. As showed in Fig. 2, an increase of PAT concentration progressively affected *Sporobolomyces* growth, which was characterized by a lag-phase at the higher concentrations of mycotoxin tested (50 and 75 μg/ml). As we previously reported, the lag-phase is longer when the concentration of PAT increases, and during this stage the mycotoxin is not actively degraded; when the yeast start growing, PAT is degraded and its decrease parallels the onset of the PAT degradation products [30]. Also a lower initial concentration of cells slowed the growth of *Sporobolomyces* in the presence of PAT and consequently its degradation. At these tested concentrations, PAT had no morphological effects on *Sporobolomyces* sp. cells as examined by microscopy.

Initially 50 μg/ml was initially chosen as the optimal PAT concentration since it has an intermediate toxicity on the cells that, however, are able to recover early from the PAT insult. For the first transcriptomic analysis, two independent *Sporobolomyces* sp. cultures were incubated in the presence and in the absence of 50 μg/ml of PAT. Cells for poly(A) RNA extraction were collected at two different growth levels, within the lag-phase at optical density 595 nm (OD_595_) ~0.08, and OD_595_ ~ 0.25 during the phase of active growth (Additional file 3A). It was expected that the transcriptomic analysis performed with the yeast growth in the lag-phase would have revealed mainly the mechanisms behind PAT resistance, while that performed during the log-phase of growth would have elucidated the specific mechanisms of PAT degradation. TLC analysis was performed to monitor the degradation process, since it was pivotal to verify that the expression of the *Sporobolomyces* sp. genes involved in PAT resistance and degradation was actually occurring. The decrease of the PAT spot progressively paralleled the increase the spots of the breakdown products, and as expected the amount of  PAT degraded was higher at OD_595_ ~ 0.25 compared to that at OD_595_ ~ 0.08. Finally, after 4 days of incubation PAT was completely degraded (Additional file 3B).

Non-strand specific sequencing was performed, which we found to be inadequate for an improvement of the existing annotation of *Sporobolomyces* genome since transcripts were merged into large gene models likely due to the overlapping UTRs of adjacent genes. This is indeed a characteristic of basidiomycete genomes, as revealed by the recent RNAseq-based genome update of the human pathogen *Cryptococcus neoformans* [39]*.* Despite this limitation, preliminary bioinformatic analysis using the *Sporobolomyces* sp. draft genome revealed that most of the upregulated genes were in common for both samples collected at two different growth stage and included more than 400 genes encoding proteins involved in oxidative and other stress responses, transport and signaling, with no evident insights about the specific mechanisms of PAT degradation (data not shown). Although informative, the large amount of data obtained and the lack of biological replicates did not allow us to focus on genes playing key functions in PAT resistance and/or degradation.

Therefore, several modifications were made to the original experimental design. First, PAT concentration was decreased from 50 μg/ml to 5 μg/ml and cells for RNA extraction were collected after 3 h of incubation, when *Sporobolomyces* sp. growth just started increasing and reached an OD_595_ value of ~ 0.08 (Additional file 4A). At this growth rate, PAT was still undegraded and ascladiols and DPA were not detected (Additional file 4B), but the genetic arsenal behind PAT resistance and degradation was active as demonstrated by the reduction of the mycotoxin and the onset of its breakdown products achieved at OD_595_ 0.8 in the same culture used for collecting the cells for analysis (Additional file 4C). The rationale behind lowering PAT concentration was to mitigate the toxic effects of the mycotoxin on the yeast cells and eliminate the lag-phase growth with the aim to detect both resistance and degradation genes involved in the first response to the mycotoxin, hence just at the beginning of the growth even before PAT degradation started. Second, strand-specific sequencing was performed in order to obtain data useful for an improvement of the genome quality, essential for a reliable transcriptomic analysis. Last, two biological replicates for a single time point rather than one replicate at different time points were used, firstly because the use of biological replicates allows for the statistical analysis of the data, and secondly because we did not see significant differences in the differentially expressed genes (DEGs) detected at different time points in our preliminary RNAseq analysis.

**Fig. 3:**
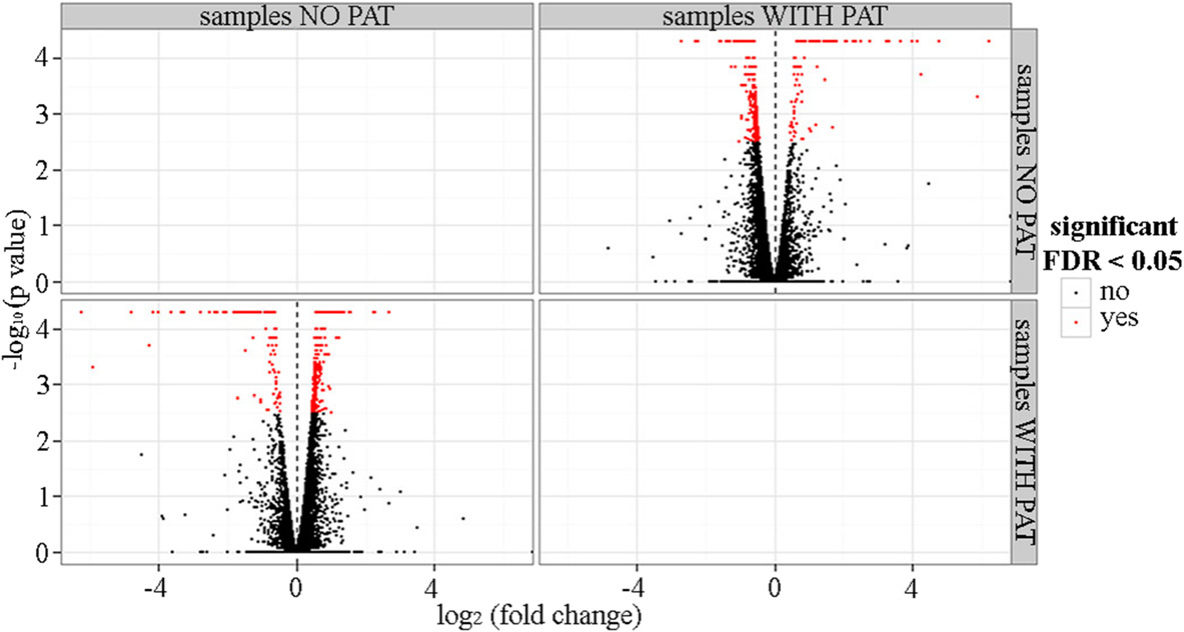
Volcano plot showing upregulated and downregulated DEGs of *Sporobolomyces* sp. IAM 13481 grown in the presence of 5 μg/ml of PAT

Bioinformatic analysis was performed using the Tuxedo tools. The identification of the *Sporobolomyces* DEGs was performed using Cuffdiff, a statistical tool within the software Cufflinks. The unprocessed Cuffdiff output is available in the Additional file 5. DEGs were considered those with a false discovery rate (FDR) < 0.05. Using these parameters, 347 genes were found differentially expressed by *Sporobolomyces* sp. IAM 13481 when incubated in the presence of 5 μg/ml of PAT. Among these, 101 were upregulated and 246 were downregulated (Fig. 3). A heat map of the expression levels of the detected DEGs is shown in the Additional file 6.

**Fig. 4:**
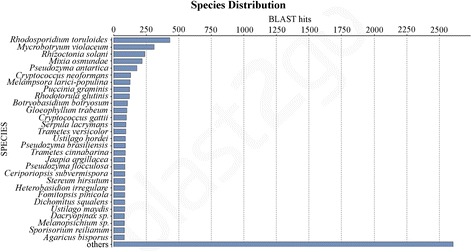
Species distribution following Blast2GO analysis of DEGs detected in *Sporobolomyces* sp. grown in the presence of 5 μg/ml of PAT

All DEGs were subjected to Blast2GO analysis (https://www.blast2go.com/b2ghome). As expected, species distribution of all BLAST hits reveals that the majority of them belong to the Basidiomycota phylum, with the most represented belonging to species in the Pucciniomycotina (*Rhodosporidium toruloides, Microbotryum violaceum, Mixia osmundae, Melampsora larici-populina, Puccinia graminis* and *Rhodotorula glutinis*), followed by the Agaricomycotina (*Rhizoctonia solani, C. neoformans*), and the Ustilaginomycotina (*Pseudozyma antartica*) (Fig. 4). DEGs were functionally annotated using the gene ontology (GO) terms and the biological process category was used for the classification. Blast2GO outcome was improved with manual integration based on the molecular function category and on the role of characterized orthologs. DEGs were used for BLASTx analysis against *S. cerevisiae* to assign gene names and to further integrate gene functions. Combined tables of the RNAseq output, *S. cerevisiae* comparison and blast2GO annotation for upregulated and downregulated genes are found in Additional files 7 and 8, respectively.

**Fig. 5:**
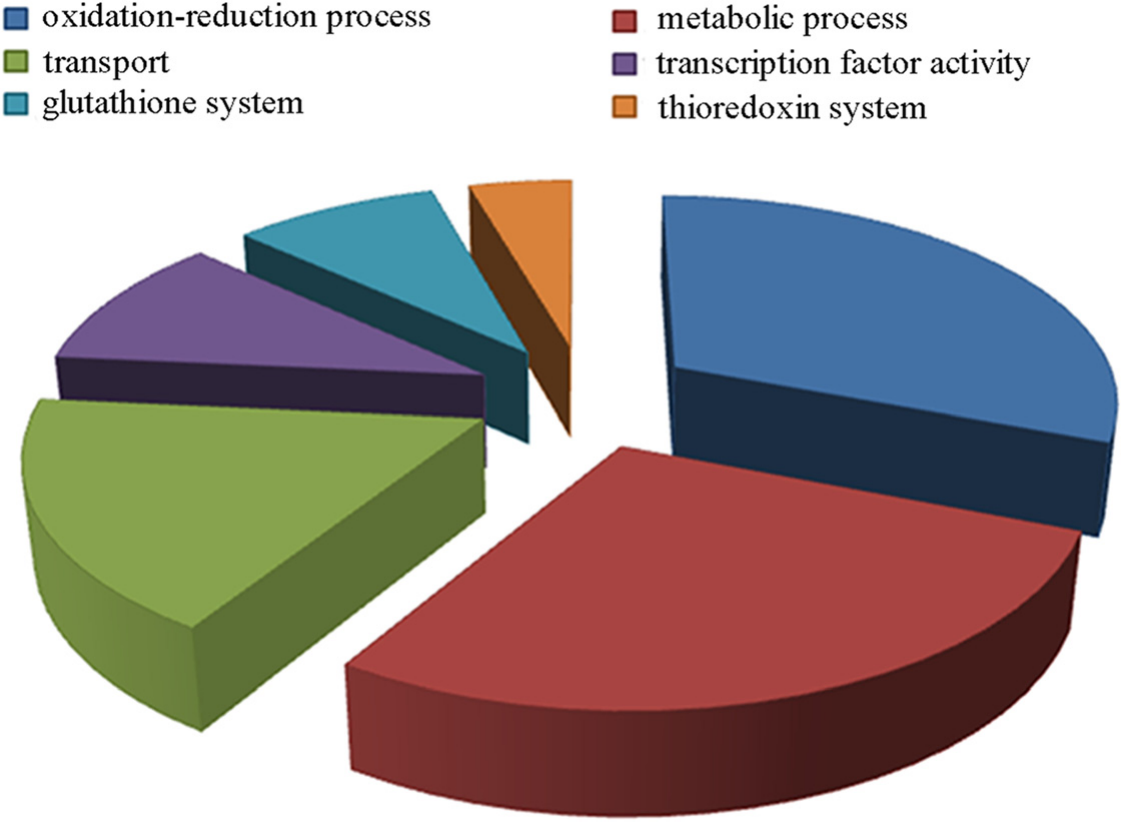
GO terms representation of upregulated DEGs of *Sporobolomyces* sp. grown in the presence of 5 μg/ml of PAT

Blast2GO analysis of upregulated DEGs assigned GO annotation for 78 genes (~77 %) that were grouped under the following GO terms: oxidation-reduction process (21 DEGs), metabolic process (19 DEGs), transport (12 DEGs), and transcription factor activity (7 DEGs). Further sub-classification of the metabolic process group found 6 DEGs involved in glutathione biosynthesis and turnover and 3 DEGs involved in the thioredoxin system. For the remaining 33 upregulated DEGs either no GO terms were assigned or they belonged to underrepresented GO term groups (Fig. 5). For gene description, the *S. cerevisiae* gene name is used; where not available, the test_id number generated by Cufflinks is used.

The DEGs in the oxidation-reduction process group included oxidases, medium and short chain dehydrogenases, and oxidoreductases that are involved in oxidative stress resistance (*OYE2, SRX1, ZTA1, ADH1, GLR1, CCP1*) and/or response to DNA replication stress (*ADH6, YML131W, GLR1, SFA1*), fatty acid beta oxidation (*SPS19*) and general stress response (*ALD5, ALD4*).

The GO group of metabolic process included DEGs encoding proteins involved in the metabolism of different cellular compartments and that play various functions in the cell. The two most expressed DEGs of this group were *ENV9* (log2FC = 4.78) and *GRE2* (log2FC = 2.35). *ENV9* encodes a NADP-binding protein proposed to be involved in vacuolar functions, and whose *S. cerevisiae* mutant shows defects in carboxypeptidase Y (CPY) processing and vacuolar morphology [40]. *GRE2* encodes a D-lactaldehyde dehydrogenase that is induced by several stresses (osmotic, ionic, oxidative, heat shock and heavy metals), it is regulated by the High Osmolarity Glycerol (HOG) pathway, and is overexpressed in response to DNA replication stress [41, 42]. Moreover, other DEGs encoded proteins involved in methionine biosynthesis (Met2, Met6 and Met28) and whose induction was also observed by Iwahashi and colleagues in *S. cerevisiae* [9], confirming that exposure to PAT activates the metabolism of the sulfur amino acid methionine likely because of its known affinity/reactivity with sulfhydryl groups [1].

Based on previous reports indicating an important involvement of glutathione [1, 9] and thioredoxin [9] in response to PAT, we specifically searched our transcriptomic data for DEGs whose function is related to these antioxidant compounds. We found 6 DEGs involved in glutathione biosynthesis and turnover and 3 of the thioredoxin system. The most upregulated glutathione gene was the *S. cerevisiae* ortholog *URE2* (log2FC = 4.15), which encodes a bifunctional protein that is involved in both nitrogen catabolite repression and oxidative stress response. Ure2 binds glutathione with high affinity and it has been shown to exhibit glutathione peroxidase activity *in vitro*; also, its crystal structure shows that it has structural similarity to glutathione *S*-transferase [43–45], which is the GO name assigned following Blast2GO analysis. Other known upregulated DEGs of the glutathione-glutaredoxin system were *GSH1, GTT2* and *GLR1*. Furthermore, our data revealed the upregulated expression of the three genes of the cytoplasmic thioredoxin system, the thioredoxins *TRX1* and *TRX2*, and the thioredoxin reductase *TRR1*.

The transport category included several genes encoding transmembrane transporters, the most expressed of which was the *FLR1*-encoding plasma membrane transporter of the major facilitator superfamily (MFS) (log2FC = 3.22). Flr1 is involved in the efflux of fluconazole, diazaborine, benomyl, methotrexate, and other drugs [46], and it was also upregulated in *S. cerevisiae* exposed to PAT [9]. Other transporter-encoding genes highly upregulated were *TPO1* (log2FC = 2.29), known to export polyamines (spermine, spermidine, and putrescine) from the cell during oxidative stress [47], the ATP binding cassette (ABC) transporters *PDR15* (log2FC = 2.61) implicated in general stress response and cellular detoxification, and the yeast cadmium factor *YCF1* (log2FC = 2.25), a vacuolar glutathione *S*-conjugate transporter required for vacuole fusion and that has a role in detoxifying metals, and transports to the vacuole glutathione disulfide (GSSG) that is not immediately reduced [48, 49].

The last group of upregulated DEGs included those encoding predicted transcription factors classified under GO molecular function terms as specific signal transducer activity and nucleic acid binding. They included two putative transcription factors whose fungal orthologs are non-characterized, and others that include three (*CRZ1, YAP1* and *SKO1*) known to activate transcription of stress response genes.

Besides the DEGs grouped under the most represented GO terms, other upregulated DEGs of interest included those encoding for a stress responsive A/B barrel domain (TCONS_00005994 log2FC = 3.99), for two retrotransposon nucleocapsid proteins (TCONS_00009558, log2FC = 3.64; TCONS_00000024, log2FC = 3.34) and for a predicted dienelactone hydrolase (TCONS_00000158, log2FC = 1.44). Last, DEGs for proteins involved in cell wall assembly and maintenance (*ECM14, EMV1*) and DNA repair (*WSS1, DDI1*) had an expression level lower than 1.

In addition to the DEGs upregulated by PAT, the cuffdiff output was also analyzed to select genes that showed expression values of FPKM (Fragments per Kilobase of Exon per Million Fragments Mapped) only in the presence of PAT. In order to avoid artifacts, genes having a FPKM value greater than 1.5 were selected (Additional file 9). GO analysis revealed that these genes encode proteins that play the same functions as those differentially upregulated, such as sulfur amino acid metabolism (Thr1), oxidation-reduction process (YDL144C, Uga2, YPR127W, Sod2 and Fre5), transport (Flr1, Git1), metabolism (Thr1, Stp14) and DNA repair (Rad53, Rad2). Of note, the overexpression of the superoxide dismutase-encoding gene *SOD1* that plays a major role in detoxification of ROS in other species was also anticipated, but BLAST analyses revealed that this gene is absent from the genome of *Sporobolomyces* sp. IAM 13481.

As regards the downregulated DEGs, Blast2GO assigned GO annotation for 145 genes (~ 59 %), with the remaining having no GO sequence description and/or GO classification. Therefore, for a more comprehensive analysis of downregulated *Sporobolomyces* DEGs in the presence of PAT, manual integration of gene classification and function was performed according to information available for the model ascomycete yeasts *S. cerevisiae* and *S. pombe.*

**Fig. 6:**
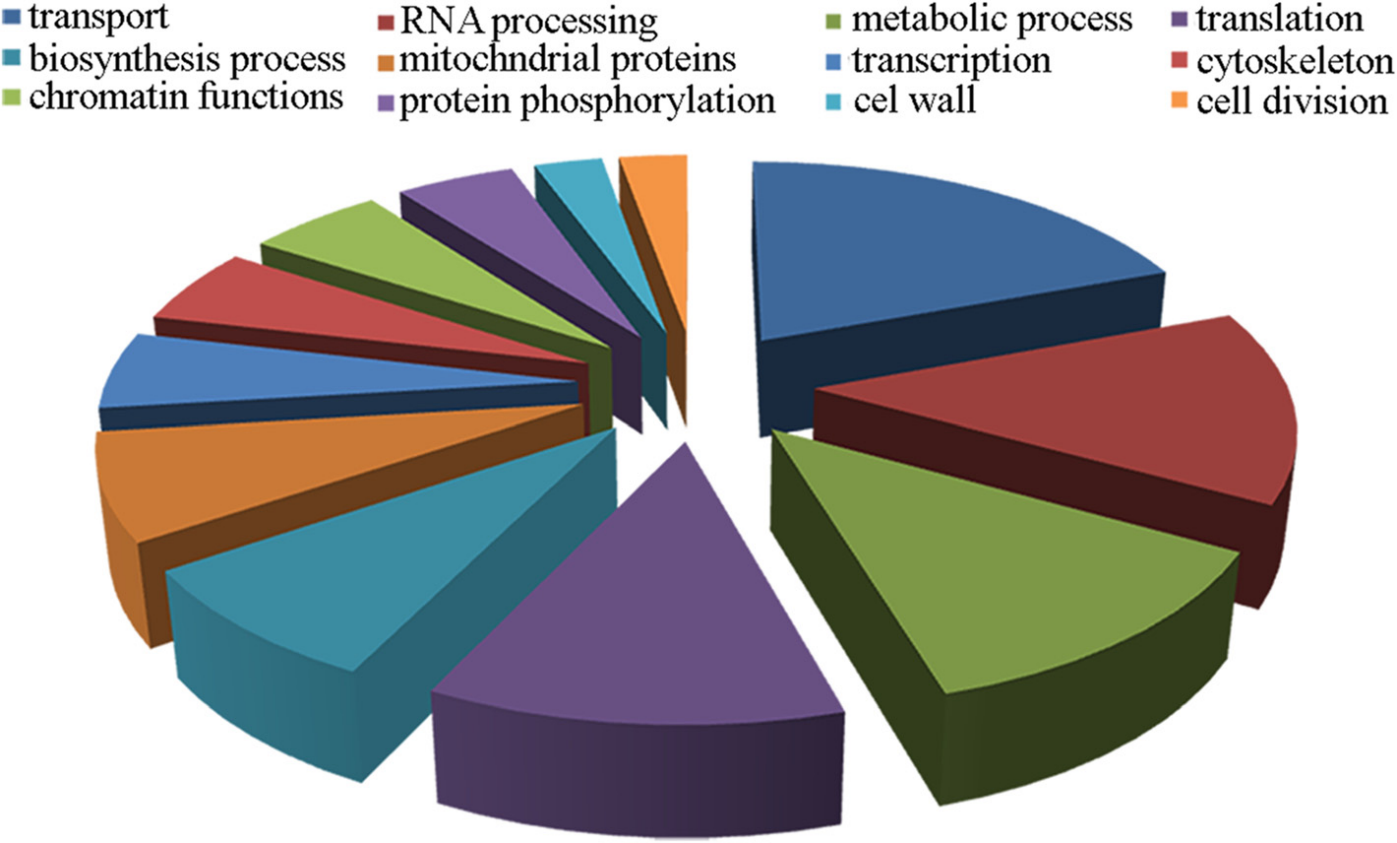
GO terms representation of downregulated DEGs of *Sporobolomyces* sp. grown in the presence of 5 μg/ml of PAT

Downregulated *Sporobolomyces* DEGs were grouped under 7 biological process categories, such as transport (28 DEGs), metabolic and biosynthesis processes (18 and 12 DEGs, respectively) and four other groups including genes that are involved in different steps of the protein synthesis process such as transcription (8 DEGs), RNA processing (20 DEGs), translation (18 DEGs) and protein phosphorylation (7 DEGs). As mentioned above, other genes were grouped according to the model yeast databases as follows: organization of the cytoskeleton (8 DEGs), cell division (4 DEGs), chromatin functions (8 DEGs), cell wall organization and function (4 DEGs) and mitochondrial functions (11 DEGs) (Fig. 6) (Additional file 8).

Downregulated DEGs of the transport group encoded i) proteins that are involved in ions and sugar homeostasis, with calcium being the most represented (YJR124C, Pmr1, Vcx1), and ii) proteins involved in vesicle-mediated transport functions from endoplasmic reticulum (ER) to Golgi, vacuole and other cellular compartments. Within the metabolic and biosynthesis groups, the most represented DEGs were those involved in amino acid biosynthesis and regulation. This result agrees with the downregulation of DEGs involved in different steps of the protein synthesis processes, which also corroborates previous findings in *S. cerevisiae* [9, 50]. Moreover, many other DEGs encode proteins involved in essential cellular processes such as organization of the cellular cytoskeleton, cell division, chromatin functions, DNA replication, meiosis, and mitosis: this suggests that cells exposed to PAT arrest their growth to have a lower metabolic activity probably because of the high energy requirements of the cells that need to recover from the insult due to the mycotoxin toxicity.

### Transcriptomic analyses of *Sporobolomyces* sp. incubated in the presence of high PAT concentration

The non-strand specific reads of poly(A) RNA extracted from *Sporobolomyces* sp. incubated in the presence of 50 μg/ml of PAT at two different growth levels (Additional file 3) were re-analyzed using the newly generated transcriptome as a reference. Because there was only one biological replicate for each time point, upregulated and downregulated DEGs were considered those having a log2FC ≥ 2 and ≤ -2, respectively. In the samples obtained at OD_595_ ~ 0.08 (indicated as PAT50#1), 155 upregulated genes were identified, and 31 of them were in common with DEGs found in samples obtained after treatment with the lower PAT concentration (indicated as PAT5); 208 downregulated DEGs with log2FC ≤ -2 were found, and 19 were in common with PAT5 (Additional file 10). Furthermore, at growth level of OD_595_ ~ 0.25 (indicated as PAT50#2) we found 204 upregulated and 188 downregulated DEGs, of which 37 and 10 were in common with PAT5, respectively (Additional file 11). Last, there was a higher similarity between the two samples exposed to 50 μg/ml PAT, with 118 upregulated and 63 downregulated DEGs in common. The transcriptional differences in cultures exposed to either 5 or 50 μg/ml of PAT may be due to the different concentrations used and to the criteria used to select DEGs, since the FDR applied to analyze biological replicates can also select genes with log2FC lower than 2 and -2.

**Fig. 7:**
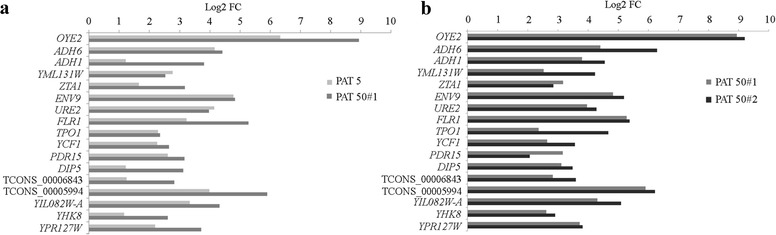
Expression comparison (expressed as Log2FC) of the main upregulated DEGs detected from *Sporobolomyces* sp. grown at the same level (OD_595_ ~ 0.08) in the presence of (**a**) 5 μg/ml (PAT5) and 50 μg/ml (PAT50#1) of PAT, or (**b**) incubated in the presence of 50 μg/ml of PAT at two different growth levels (OD_595_ ~ 0.08, PAT 50#1; OD_595_ ~ 0.25, PAT 50#2)

Amongst the three different transcriptomic datasets of this study (PAT5, PAT50#1 and PAT50#2), some of the most upregulated DEGs in all the samples were selected and their expression levels compared at different PAT concentrations and at two different growth stages (Fig. 7). As expected, at a similar growth level (i.e. OD_595_ ~ 0.08) an increase of PAT concentration from 5 to 50 μg/ml led to a higher expression of all the DEGs selected, with the exception of YML131W and *URE2*. Also, for the majority of the selected genes there was a minimum of a one-unit log2FC increment of expression at 50 μg/ml, indicating that the abundance of transcripts for proteins such as Oye2, Adh1, Flr1, and others, relate to PAT toxicity and thus specifically required to defeat the cellular insult caused by the mycotoxin. Conversely, the expression of other genes (*ADH6,* YML131W*, ENV9, URE2, TPO1, YCF1* and *PDR15*) did not considerably differ at the two tested PAT concentrations, suggesting that the encoded proteins might be involved in general response to a toxic molecule (Fig. 7a). Furthermore, with the exception of *PDR15*, there was a higher expression of all the genes selected during the growth of *Sporobolomyces* in the presence of 50 μg/ml of PAT (Fig. 7b), suggesting that the mechanisms to overcome PAT toxicity might be active and increase during the degradation process while PAT is still present in the medium.

Real time reverse transcriptase PCR was used to validate the expression levels of a subset of common genes identified in the three transcriptomic datasets. The relative expression levels of *OYE2, ENV9, URE2, FLR1*, TCONS_00006843 and TCONS_00005994 by PAT treatment were tested. All six genes had high transcript levels in response to different patulin concentrations, confirming the regulation of these genes defined by the RNAseq data (data not shown).

## Discussion

**Fig. 8:**
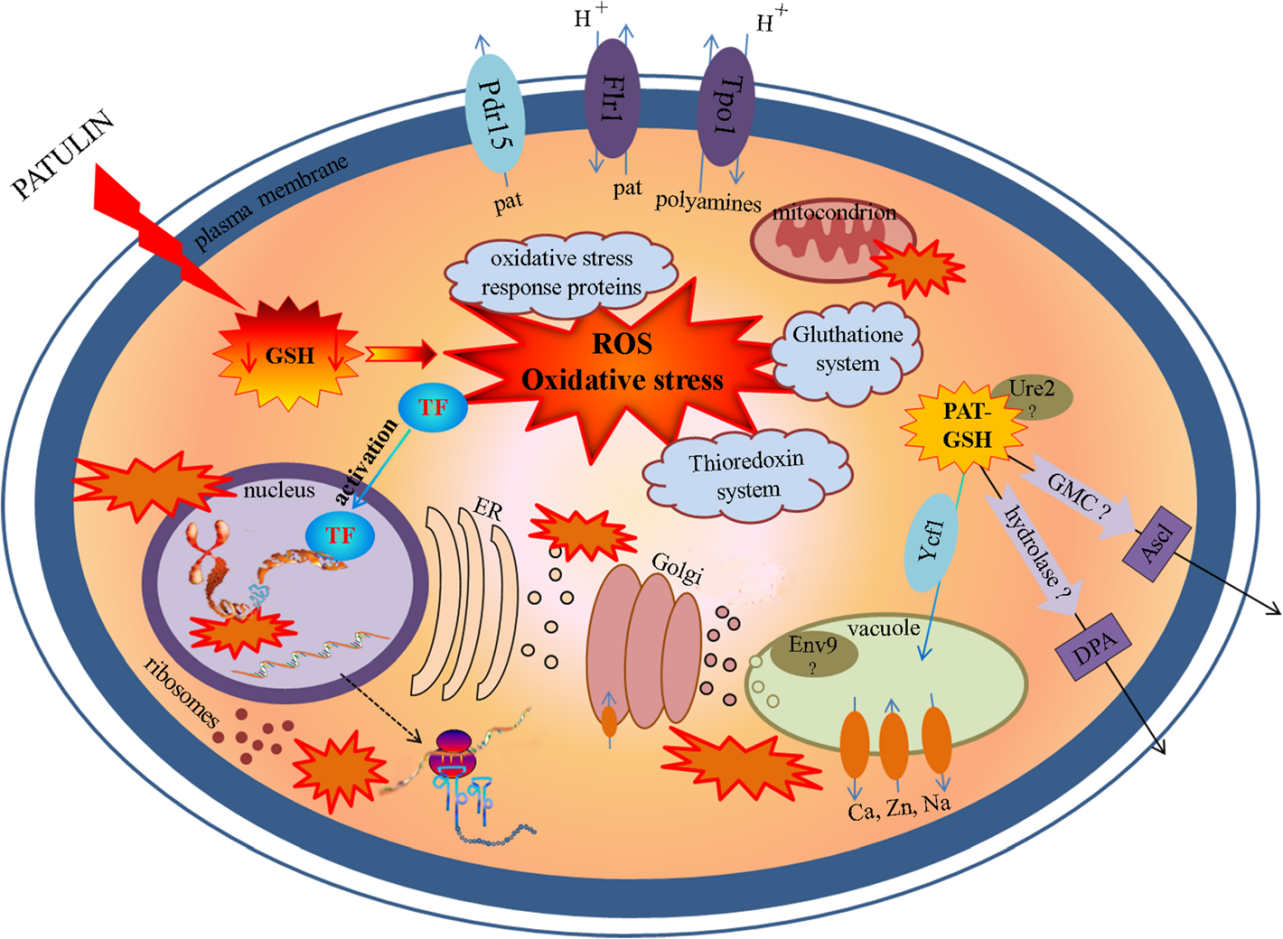
Model for *Sporobolomyces* sp. responses to the mycotoxin PAT. The model is based on information found in the present study and in the existing literature (see Discussion for details). The explosion shapes indicate oxidative damages. GSH is gluthatione; PAT-GSH is glutathione adduct with PAT mediated by Ure2; TF is transcription factor; ER is endoplasmic reticulum. The question marks indicate an unknown function/process

By combining the transcriptomic data obtained in the present study with the previous experiments primarily in *S. cerevisiae*, we provide an overview of the global effects of PAT on *Sporobolomyces* sp. and other eukaryotes (Fig. 8). The genera *Sporobolomyces* and *Saccharomyces* diverged an estimated 800–1600 million years ago, with those estimates based on different molecular clock calibrations [51]. Hence, observing a common set of differentially-regulated genes that are shared over such long time frames could indicate key components regulated in response to this mycotoxin.

### Effects of PAT on cells

At a cellular level, PAT has a high electrophilic reactivity with sulfhydryl group-containing compounds [4, 52]. The mycotoxin interacts with the cell wall and targets free thiols of the outer surface of the plasma membrane [14, 53] thus compromising its permeability and integrity. This, in conjunction with the low molecular weight of PAT, allows the mycotoxin to enter the cell, most likely through passive diffusion without the involvement of transporters or energy-dependent pumps. A significant role in plasma membrane destabilization is likely due also to lipid peroxidation caused by the oxidative burst described below.

Once in the cell, PAT causes GSH depletion through reaction with its sulfhydryl groups; this results in the alteration of the cellular redox homeostasis [5, 6]. We suggest that this is the key event for PAT cytotoxicity as it results in an increased generation of ROS and a subsequent oxidative burst that induces early transmission of stress signals and is responsible for the damage to cellular components. As highlighted in the following description, this hypothesis is based on the transcriptional response of *S. cerevisiae* to PAT [9, 35] as well as with ROS-mediated stress response studies in *S. cerevisiae* and *C. neoformans* [11, 12, 54].

#### Transmission of stress signals

Based on a number of lines of evidence, hydrogen peroxide (H_2_O_2_) is a major ROS involved in signaling responses and is produced by NAPDH oxidases in conjunction with superoxide dismutase [11, 12, 54]. We suggest that the *Sporobolomyces* sp. adaptive response to oxidative stress generated by PAT is regulated by the transcription factors identified in our analysis, which in conjunction activate the expression of stress-response proteins, and antioxidant and detoxification systems. Of note, it is likely that an important role is played by Yap1, since this bZIP transcription factor is a key component in H_2_O_2_-mediated signaling transduction in *S. cerevisiae* [11, 12] where it is overexpressed also following PAT treatment [9, 35]; other *S. cerevisiae* transcription factors upregulated following PAT and ROS exposure are Skn7, Msn2 and Msn4. However, they were not identified in our study, suggesting that the response of *Sporobolomyces* and *S. cerevisiae* to ROS involves different transcription factors, with the exception of Yap1.

#### Activation of stress responsive proteins

Genes of *Sporobolomyces* sp. encoding cytoplasmic (Oye2, Adh6, Gre2, Yml131w, Sps19, Zta1, Adh1, etc.) and mitochondrial proteins (Oye2, Sod2, Ald4, Ald5, Sfa1, Erv1, etc.) that are upregulated following oxidative stress are designed with the GO molecular function term “oxidoreductase activity”. Interestingly, the majority of these stress responsive genes were overexpressed in *S. cerevisiae* also in response to DNA damaging-agents [42], implicating oxidative damage to DNA caused by PAT as previously reported in other studies [6, 55, 56].

#### Activation of antioxidant systems

In concordance with the findings of Iwahashi et al. in *S. cerevisiae* [9], our analysis revealed that the most important increase in transcript levels was in *Sporobolomyces* genes associated with the antioxidant molecules glutathione and thioredoxin. However, as regard the glutathione cycle, we only found upregulation of *URE2*, *GSH1* and *GLR1*, while in *S. cerevisiae* Iwahashi and colleagues found also *GRX2* and *PRX1* that encode respectively the cytoplasmic glutaredoxin and the mitochondrial thioredoxin. Also, we did not detect the genes encoding for the antioxidant proteins Tsa1, Tsa2, Ahp1, Ahp2 (peroxiredoxins) and Hyr1 (thiol peroxidase). Another important scavenger system activated by *Sporobolomyces* in response to PAT is the thioredoxin one: on the basis of our transcriptomic data, this system involves mainly the three cytoplasmic proteins Trr1, Trx1 and Trx2. In *S. cerevisiae* the thioredoxin system is preceded by the peroxiredoxin cycle [11] whose components (Tsa1, Tsa2, Ahp1 and Ahp2) were overexpressed also in *Sporobolomyces* sp. exposed to PAT, although not statistically significant according to Cuffdiff. These antioxidant systems are well conserved amongst fungi and their function in *Sporobolomyces* sp. is likely the same as in *S. cerevisiae* [11] and in the basidiomycete yeast *C. neoformans* in which they have been characterized [57].

#### Export and detoxification proteins

Another mechanism mediated by the transcription factors in response to the oxidative stress caused by PAT is the activation of genes encoding transmembrane transporters involved in export of metabolites from the cell. The main transporters identified were the MFS member Flr1 and the ABC transporter Pdr15. We suggest that these proteins are involved in PAT efflux in a similar way as occurs in the multidrug resistance in fungi and bacteria. Also, the MFS transporter Tpo1 likely exports polyamines during PAT-induced oxidative stress, as suggested for *S. cerevisiae* [47].

#### Mechanisms of PAT degradation

PAT is degraded in *Sporobolomyces* sp. through two independent pathways that lead to the formation of DPA and ascladiols [30]. The same PAT breakdown products have been identified in other Pucciniomycotina yeasts such as *R. kratochvilovae* [28, 29], *R. paludigenum* [31] and *Rhodotorula mucilaginosa* (our unpublished data). Following our transcriptomic analysis we suggest that PAT degradation occurs through different ways and an essential step is the formation of enzymatic adducts of PAT with GSH, as documented by Fliege and Metzler [4]. We propose that the degradation occurs at least in part in the vacuole, likely mediated by the ABC transporter Ycf1 that binds PAT-GSH adducts and actively transports them to the vacuole for non-specific degradation; other vacuolar proteins, such as Env9, may also be involved. This GSH mechanism controls resistance of *S. cerevisiae* to the oxidative stress-inducing heavy metals cadmium, nickel, arsenic and mercury [58–60]. Of note, the enzymatic adducts are likely formed by proteins with glutathione *S*-transferase activity [4] such as Ure2, which is one of the highly expressed DEGs in our analysis. Whether or not the formation of the PAT breakdown products DPA and ascladiol occurs in the vacuole, other organelles, or in the cytosol needs to be further investigated.

DPA is the most important metabolite of PAT degradation because i) it is characterized by a lower cytotoxicity than PAT, most likely because of its very low reactivity with thiol groups [29], ii) it is the most abundant breakdown metabolite, and iii) it is produced by all the Pucciniomycotina found as able to degrade PAT. DPA is most likely formed through the hydrolysis of the α,β-unsaturated γ-lactone ring of PAT (Rosa Durán-Patrón, personal communication), a mechanism that is similar to the biodetoxification of zearalenone by the zearalenone hydrolase/lactonase Zdh101 produced by the BCA *Clonostachys rosea* [61]. Orthologs of Zdh101 were not found amongst the DEGs identified in this study as well as in *Sporobolomyces* and other sequenced Pucciniomycotina genomes. Therefore, we searched for genes encoding proteins with predicted hydrolase activity on lactone rings and found in our DEGs a predicted dienelactone hydrolase (TCONS_00000158). We previously generated a targeted mutant for this gene as well as for another gene encoding a dienelactone hydrolase, the latter not detected as being differentially expressed in our analysis. Surprisingly, both mutants had the same kinetics of PAT degradation as the *Sporobolomyces* wild type strain (unpublished data), indicating that these genes are not involved in PAT degradation. Perhaps suitable candidates for DPA generation could be the newly identified gene (TCONS_00005994) that encodes a protein containing a stress responsive A/B barrel domain of unknown function, another gene (TCONS_00013529) that encodes a protein designed as a catalytic LigB subunit of aromatic ring-opening dioxygenase (TCONS_00013529), or one of the upregulated short or medium chain dehydrogenases that are potentially able to degrade xenobiotic compounds.

As regards ascladiol, from a chemical viewpoint it results from the hemiacetal opening of PAT and reduction of the derived aldehyde. *(E)-*ascladiol is oxidized to PAT in one step enzymatic reaction in PAT biosynthesis [62]; thus, it is reasonable that the reverse enzymatic reaction leads to the formation of *(E)-*ascladiol from PAT. It has been recently suggested that the *P. expansum* gene *PatE* encoding a Glucose-methanol-choline (GMC) oxidoreductase is responsible for the last step in PAT biosynthesis [22], even though this hypothesis has not been experimentally established yet. Strikingly, BLASTp analysis revealed that *P. expansum PatE* corresponds to the *Sporobolomyces* GMC oxidoreductase that was found to be upregulated in the present study (TCONS_00004874), and potentially this enzyme may be responsible for the conversion of PAT into ascladiol. Future studies using reverse genetics will allow the elucidation of the role of the upregulated *Sporobolomyces* sp. DEGs in PAT resistance and degradation.

#### Damage to cellular components

Besides the role of ROS as signaling molecules, they are mainly responsible for PAT cytotoxicity. In fact, despite the rapid upregulation and high efficiency of antioxidant defenses, cells can still sustain severe organelle damage and inhibition of biological processes essential for cellular life. The cytotoxic effects of PAT have been studied for over 30 years, as reviewed in 2005 and 2010 [1, 2], and they recall the cellular damage observed in *S. cerevisiae* exposed to ROS [12]. Lipid peroxidation causes perturbing effects on the plasma and other organelle membranes with loss of intracellular components and altered homeostasis. Furthermore, oxidative DNA and RNA damage compromises nucleic acids integrity and functionality hence interfering with their synthesis and replication; the actin cytoskeleton is another target of PAT as well as of ROS, and the physiological consequences of its oxidation include accelerated aging and apoptotic cell death [63].

## Conclusions

This is the first study where a next generation sequence (NGS) technique has been applied to depict the complex molecular effects of the mycotoxin PAT on a non-conventional basidiomycete yeast that is able to degrade it and has biocontrol activity against the PAT-producing fungus *P. expansum*. Our study when compared with previous findings from *S. cerevisiae* about PAT cytotoxicity provides compelling evidence for common cellular targets and responses, yet it also discloses new aspects such as novel mechanisms of stress response activation, which may represent a specialized feature of the Pucciniomycotina fungi. Two attractive long term goals are 1) the development of a biosensor, based on upregulated *Sporobolomyces* and *S. cerevisiae* genes in response to or even that bind the mycotoxin, for user-friendly and economical procedures for PAT detection and quantification in products ready for commercialization; and 2) the functional characterization of genes involved in conversion of PAT to DPA and/or ascladiol that will create the premises for production of enzymes responsible for PAT detoxification in heterologous cell factories in which the identified genes are transformed. These enzymes could be the base for detoxification processes of products and juices derived from pome fruits.

## Methods

### Strain used and cultivation

*Sporobolomyces* sp. strain IAM 13481 was used in this study. The strain was routinely cultivated on yeast peptone dextrose medium (YPD: Yeast extract 10 g/l, Peptone 20 g/l, D-glucose 20 g/l, Agar 20 g/l). *Sporobolomyces* sp. was incubated at concentration of 1×10^7^ CFU/ml in Lilly Barnett medium (LiBa, 10.0 g D-glucose, 2.0 g L-asparagine, 1.0 g KH_2_PO_4_, 0.5 g MgSO_4_ · 7H_2_O, 0.01 mg FeSO_4_ · 7H_2_O, 8.7 mg ZnSO_4_ · 7H_2_O, 3.0 mg MnSO_4_ · H_2_O, 0.1 mg Biotin, and 0.1 mg Thiamine, per Liter) [64] supplemented with 5, 15, 30, 50 and 75 μg/ml of PAT (A.G. Scientific, Inc.; San Diego, CA, USA), and the growth monitored on a daily basis by reading the OD_595_ in a microplate reader. Transcriptomic analysis was performed for *Sporobolomyces* sp. incubated in the presence of 5 μg/ml and 50 μg/ml of PAT. With 5 μg/ml of PAT, two biological replicates were used; *Sporobolomyces* sp. cells were added to 200 ml of LiBa medium and incubated on an orbital shaker at 24 °C until the growth reached a OD_595_ of ~ 0.08 (~3 h). The untreated cultures were *Sporobolomyces* sp. incubated in LiBa without PAT at the same growth level. The cultures were centrifuged to collect cells for RNA extraction, and an aliquot of each supernatant obtained was analyzed through HPLC as previously described [30] to monitor the degradation of PAT at the time of extraction. When 50 μg/ml of PAT was used, two independent *Sporobolomyces* sp. cultures of 50 ml were incubated on an orbital shaker at 24 °C and cells for RNA extraction were collected by centrifugation at OD_595_ of ~ 0.08 from one culture, and at OD_595_ of ~ 0.25 from the other; untreated conditions were LiBa medium without PAT inoculated with *Sporobolomyces* sp. IAM 13481 reaching the same growth levels. In this case the supernatants obtained were used to assess through TLC analysis [30] the degradation of PAT at the times of RNA extraction. Moreover, both at PAT 5 and 50 μg/ml, aliquots of the growth cultures from which cells were collected for RNA extraction were re-incubated and used to assess *Sporobolomyces* growth and PAT degradation for several days, since it was pivotal to verify that the mechanisms behind PAT resistance and degradation were active.

Besides the incubation in the presence of PAT, *Sporobolomyces* sp. RNA was extracted also from cells collected on YPD agar after two days of growth, and from fired ballistospores collected from a mirror YPD plate [37]; in the present study these data were only used for the generation of the *Sporobolomyces* sp. reference transcriptome (Additional file 1). Collected yeast cells were frozen, lyophilized and stored at -80 °C until RNA extraction.

### RNA extraction and Illumina sequencing

Total RNA was extracted from *Sporobolomyces* sp. cells with the Trizol reagent according to the manufacturer’s instruction (Invitrogen, Grand Island, NY). Quality and concentration of total RNA was checked by gel electrophoresis and a spectrophotometer, respectively. Libraries construction and Illumina sequencing were performed at the DNA Core Facility of the University of Missouri-Columbia. For RNA samples obtained from *Sporobolomyces* sp. cells incubated in the presence of 50 μg/ml of PAT and respective controls, TruSeq non-strand specific libraries were prepared. For RNA samples extracted from *Sporobolomyces* sp. cells incubated in the presence of 5 μg/ml of PAT and from a mirror plate, including their respective controls, TruSeq strand-specific libraries were prepared since this method allows a more accurate generation of a reference transcriptome (Additional file 1). In both cases, 100 bp single end reads were sequenced using an Illumina HiSeq2000 instrument.

### Bioinformatic analyses of the expression of the RNAseq libraries

Bioinformatic analyses were performed using the Tuxedo tools [38]. First, the existing *Sporobolomyces* sp. genome annotation was examined using the RNAseq data presented in this study. To this aim, a total of six strand-specific libraries generated as reported above were used. The *Sporobolomyces* sp. genome sequence (.fasta format) and annotation (.gtf format) were downloaded from the US Department of Energy’s Joint Genome Institute website (http://genome.jgi-psf.org/Sporo1/Sporo1.home.html). Cleaned reads generated from all six conditions were individually mapped to the *Sporobolomyces* sp. genome sequence using Tophat. Generated .bam files were used to run Cufflinks with the RABT (Reference Annotation Based Transcript) assembly, an option that it is able to integrate novel transcripts in the existing genome annotation. Cuffmerge was then used to merge all the .gtf files generated by Cufflinks into the reference transcriptome used in downstream differential analysis. Novel *Sporobolomyces* sp. genes were those that did not find a corresponding match in the JGI *Sporobolomyces* sp. genome annotation. Gene names were assigned according to the Saccharomyces genome database (SGD) following BLASTx analysis; matches with *E*-value higher than 1E-3 were not considered because they indicated different orthologs having only low similarity. Cuffdiff, the statistical software for gene expression analysis, was run to compare the Tophat outputs deriving from samples of *Sporobolomyces* sp. incubated with and without 5 μg/ml of PAT (Additional file 1), and with the newly generated transcriptome as reference. *Sporobolomyces* sp. DEGs were considered those with a FDR < 0.05. Graphical representations were performed using CummeRbund within the R package. Functional annotation of *Sporobolomyces* sp. DEGs was performed using the Blast2GO pipeline, which includes the BLASTx against the NCBI non-redundant protein database, gene ontology (GO) annotation and InterProScan [65].

Non-strand sequences generated from samples of *Sporobolomyces* sp. incubated with or without 50 μg/ml of PAT at two time points were analyzed using Tophat-Cufflinks as described above; the newly generated strand-specific based reference transcriptome was used. Because samples were as single biological replicates, DEGs were selected based on the Log2FC expression values, with those ≥ 2 being upregulated and those ≤ -2 downregulated. DEGs selected were compared with those obtained for *Sporobolomyces* sp. incubated with 5 μg/ml of PAT, as indicative of the reliability of our analysis since these datasets originated from three different and independent conditions. Furthermore, in order to evaluate the genetic effects of the PAT concentration and the incubation time, the Log2FC expression values of the main upregulated DEGs of these three datasets were compared.

### Availability of supporting data

All the supporting data are included as Additional file.

## Additional files

Additional file 1: Table reporting growth conditions used to collect *Sporobolomyces* samples for RNA extraction, the type of cDNA libraries generated, and their use for downstream analysis. (DOCX 79 kb) Additional file 2: All new assembled *Sporobolomyces* sp. isoforms used for BLAST analyses. (TXT 34275 kb) Additional file 3: (A) Growth of *Sporobolomyces* sp. in LiBa medium in the presence and absence of 50 μg/ml of PAT. The arrows indicate when *Sporobolomyces* sp. cells were collected for RNA extraction. (B) TLC analysis of the growth medium from which *Sporobolomyces* sp. cells were collected for RNA extraction. The lanes with asterisks correspond to the time when *Sporobolomyces* sp. cells were collected (i.e. grey arrows). PAT = patulin; DPA = desoxypatulinic acid; ASCL = ascladiol. TLC analysis was performed until no more PAT was detected. (TIF 749 kb) Additional file 4: (A) Growth of *Sporobolomyces* sp. in LiBa medium in the presence and absence of 5 μg/ml of PAT, with the first five hours showed in detail in the small box. The arrows indicate when *Sporobolomyces* sp. cells were collected for RNA extraction. (B) HPLC analysis showing an overlay between the growth medium containing *Sporobolomyces* cells with PAT 5 μg/ml at the time of RNA extraction, and a cell-free LiBa plus PAT 5 μg/ml; note that the PAT peaks have the same area, indicating that PAT degradation had not started yet. (C) HPLC chromatograms of the growth medium from which *Sporobolomyces* cells were collected for RNA extraction after 48 h of incubation (OD_595_ ~ 0.8). As already known, only *(Z)*-ascladiol and DPA were clearly detected as the final products of PAT degradation by *Sporobolomyces*. (TIF 124 kb) Additional file 5: Unprocessed Cuffdiff output of the RNAseq experiments performed for *Sporobolomyces* grown in the presence of 5 μg/ml of PAT. Where no *Sporobolomyces* gene models are indicated based on the previous JGI annotation (column C), it indicates a novel transcript identified following the transcriptomic analysis presented in this study. (XLSX 1622 kb) Additional file 6: Heat map showing upregulated and downregulated DEGs of *Sporobolomyces* sp. grown in the presence of 5 μg/ml of PAT. (TIF 22235 kb) Additional file 7: Combined table of cuffdiff, Blast2GO and BLASTx outputs for upregulated *Sporobolomyces* sp. DEGs (FDR < 0.05) detected in the presence of 5 μg/ml of PAT. DEGs are grouped based on GO term classification, and within the groups sorted for log2 FC values (from the highest to the lowest). BLASTx results with existing *Sporobolomyces* sp. genome annotation were useful to identify new genes detected in the present study, while BLASTx against SGD was necessary to identify *Sporobolomyces* sp. orthologs in *S. cerevisiae*; low homology (i.e. *E*-values positive or higher than 1E-3) was reported as NA indicating that no *S. cerevisiae* orthologs of *Sporobolomyces* sp. DEGs were found. (XLSX 27 kb) Additional file 8: Combined table of cuffdiff, Blast2GO and BLASTx outputs for downregulated *Sporobolomyces* sp. DEGs (FDR < 0.05) detected in the presence of 5 μg/ml of PAT. DEGs are grouped based on GO term classification and within the groups sorted for log2 FC values (from the lowest to the highest). BLASTx results with existing *Sporobolomyces* sp. genome annotation were useful to identify new genes detected in the present study, while BLASTx against SGD was necessary to identify *Sporobolomyces* sp. orthologs in *S. cerevisiae* and accordingly assign gene names; low homology (i.e. *E*-values positive or higher than 1E-3) was reported as NA indicating that no *S. cerevisiae* orthologs of *Sporobolomyces* sp. DEGs were found. (XLSX 54 kb) Additional file 9: Combined table of cuffdiff, Blast2GO and BLASTx outputs for *Sporobolomyces* sp. genes detected only in the presence of 5 μg/ml of PAT. DEGs have been sorted for FPKM expression values (from the highest to the lowest), and those with FPKM lower than 1.5 were discarded. BLASTx results with existing *Sporobolomyces* sp. genome annotation were useful to identify new genes detected in the present study, while BLASTx against SGD was necessary to identify *Sporobolomyces* sp. orthologs in yeast and to assign gene names accordingly; low homology (i.e. *E*-values positive or higher than 1E-3) was reported as NA, indicating that no *S. cerevisiae* orthologs of *Sporobolomyces* sp. DEGs were found. (XLSX 13 kb) Additional file 10: Combined table of cuffdiff and BLASTx outputs for upregulated (Log2FC ≥ 2) and downregulated (Log2FC ≤ -2) DEGs detected for *Sporobolomyces* sp. grown in the presence of 50 μg/ml of PAT at OD_595_ of ~0.08. BLASTx against existing *Sporobolomyces* sp. genome annotation and SGD is also shown. DEGs have been sorted from the highest Log2FC value to the smallest. In bold are indicated DEGs found also for *Sporobolomyces* sp. incubated in the presence of 5 μg/ml of PAT reported in additional files 7 and 9. (XLSX 52 kb) Additional file 11: Combined table of cuffdiff and BLASTx outputs for upregulated (Log2FC ≥ 2) and downregulated (Log2FC ≤ - 2) DEGs detected for *Sporobolomyces* sp. grown in the presence of 50 μg/ml of PAT at OD_595_ of ~0.25. BLASTx against existing *Sporobolomyces* sp. genome annotation and SGD is also shown. DEGs have been sorted from the highest Log2FC value to the smallest. In bold are indicated DEGs found also for *Sporobolomyces* sp. incubated in the presence of 5 μg/ml of PAT reported in Additional files 7 and 9. (XLSX 58 kb)

## Abbreviations

ABC: ATP binding cassette
BCA: biocontrol agent
CPY: carboxypeptidase Y
DEGs: differentially expressed genes
DPA: desoxypatulinic acid
EC: European Commission
ER: endoplasmic reticulum
FDR: false discovery rate
FPKM: fragments per kilobase of exon per million fragments mapped
GMC: glucose-methanol-choline
GO: gene ontology
GSH: glutathione
GSSG: glutathione disulfide
H_2_O_2_: hydrogen peroxide
HOG: high osmolarity glycerol
MFS: major facilitator superfamily
NGS: next generation sequence
OD: optical density
PAT: patulin
RABT: reference annotation based transcript
RNAseq: RNA sequencing
ROS: reactive oxygen species
SGD: Saccharomyces genome database
SRA: sequence read archive
UTRs: untranslated regions
