# Supplementary material for: Transcriptomic responses of the basidiomycete yeast Sporobolomyces sp. to the mycotoxin patulin
Source: BMC Genomics. 2016 Mar 9;17:210. doi: 10.1186/s12864-016-2550-4 (PMC4784387; doi:10.1186/s12864-016-2550-4)
Supplement: Additional file 1: — Table reporting growth conditions used to collect Sporobolomyces samples for RNA extraction, the type of cDNA libraries generated, and their use for downstream analysis. (DOCX 79 kb) [file 12864_2016_2550_MOESM1_ESM.docx]

| **Samples for RNA extraction** | **Type of library** | **Downstream analysis** |
| --- | --- | --- |
| *Sporobolomyces* sp. in LiBa - OD_595_ ~ 0.08 - replicate I | TruSeq strand-specific | - Reference transcriptome generation  - Analysis of DEGs |
| *Sporobolomyces* sp. in 5 µg/ml of PAT - OD_595_ ~ 0.08 - replicate I | TruSeq strand-specific | - Reference transcriptome generation  - Analysis of DEGs |
| *Sporobolomyces* sp. in LiBa - OD_595_ ~ 0.08 - replicate II | TruSeq strand-specific | - Reference transcriptome generation  - Analysis of DEGs |
| *Sporobolomyces* sp. in 5 µg/ml of PAT - OD_595_ ~ 0.08 - replicate II | TruSeq strand-specific | - Reference transcriptome generation  - Analysis of DEGs |
| *Sporobolomyces* sp. from YPD agar | TruSeq strand-specific | - Reference transcriptome generation |
| *Sporobolomyces* sp. ballistospores fired on a YPD agar mirror plate | TruSeq strand-specific | - Reference transcriptome generation |
| *Sporobolomyces* sp. in LiBa - OD_595_ ~ 0.08 | TruSeq non-stranded | - Analysis of DEGs using the newly generated reference transcriptome |
| *Sporobolomyces* sp. in 50 µg/ml of PAT - OD_595_ ~ 0.08 | TruSeq non-stranded | - Analysis of DEGs using the newly generated reference transcriptome |
| *Sporobolomyces* sp. in LiBa - OD_595_ = 0.2 | TruSeq non-stranded | - Analysis of DEGs using the newly generated reference transcriptome |
| *Sporobolomyces* sp. in 50 µg/ml of PAT - OD_595_ ~ 0.2 | TruSeq non-stranded | - Analysis of DEGs using the newly generated reference transcriptome |
