# Supplementary figures and images for: Transcriptomic responses of the basidiomycete yeast Sporobolomyces sp. to the mycotoxin patulin
Source: BMC Genomics. 2016 Mar 9;17:210. doi: 10.1186/s12864-016-2550-4 (PMC4784387; doi:10.1186/s12864-016-2550-4)

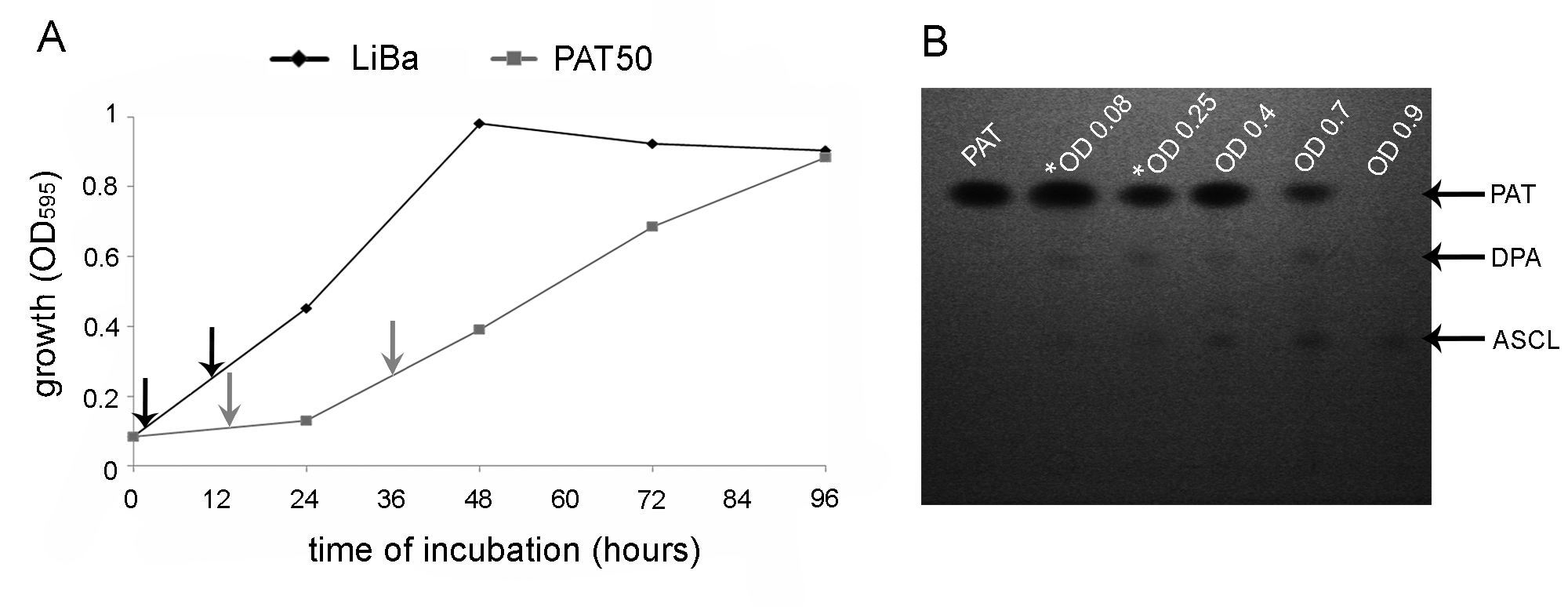

Supplement: Additional file 3: — (A) Growth of Sporobolomyces sp. in LiBa medium in the presence and absence of 50 μg/ml of PAT. The arrows indicate when Sporobolomyces sp. cells were collected for RNA extraction. (B) TLC analysis of the growth medium from which Sporobolomyces sp. cells were collected for RNA extraction. The lanes with asterisks correspond to the time when Sporobolomyces sp. cells were collected (i.e. grey arrows). PAT = patulin; DPA = desoxypatulinic acid; ASCL = ascladiol. TLC analysis was performed until no more PAT was detected. (TIF 749 kb) [file 12864_2016_2550_MOESM3_ESM.tif]

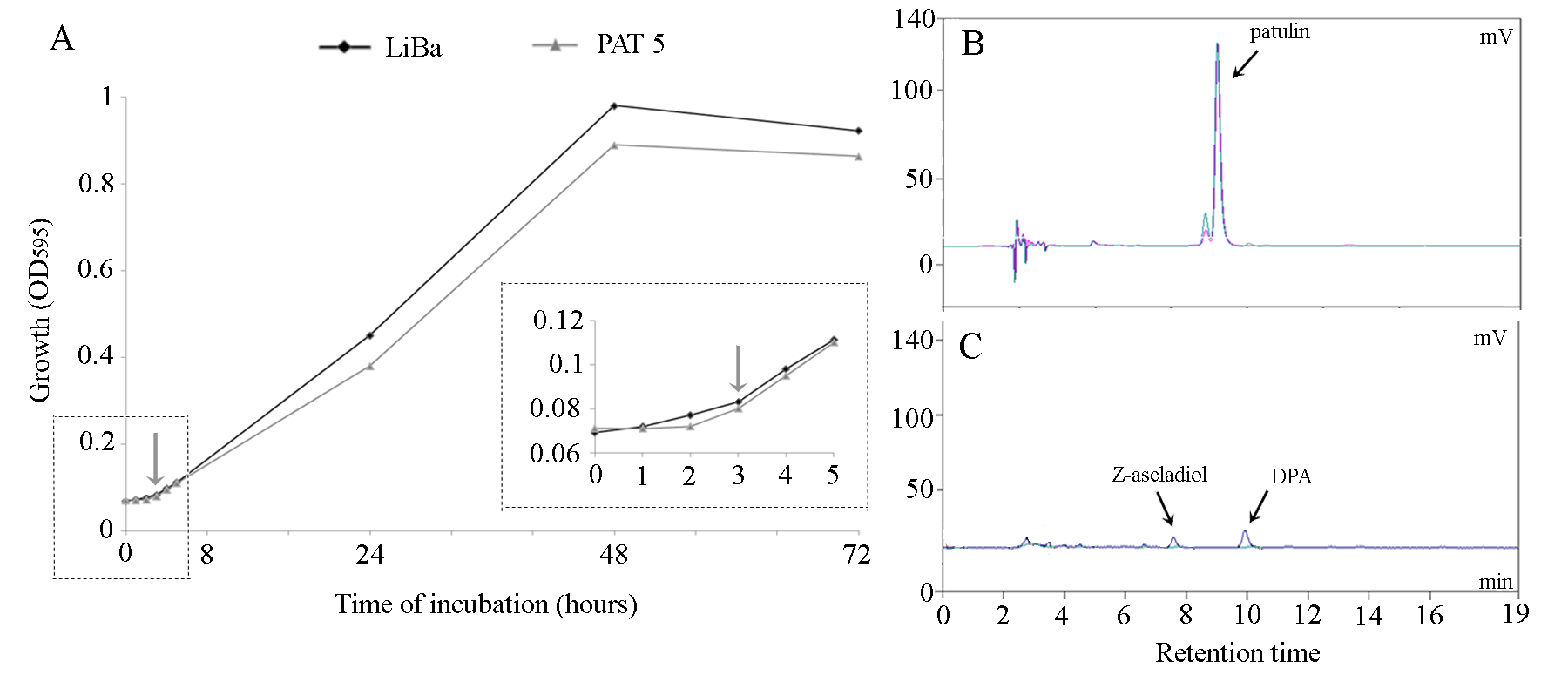

Supplement: Additional file 4: — (A) Growth of Sporobolomyces sp. in LiBa medium in the presence and absence of 5 μg/ml of PAT, with the first five hours showed in detail in the small box. The arrows indicate when Sporobolomyces sp. cells were collected for RNA extraction. (B) HPLC analysis showing an overlay between the growth medium containing Sporobolomyces cells with PAT 5 μg/ml at the time of RNA extraction, and a cell-free LiBa plus PAT 5 μg/ml; note that the PAT peaks have the same area, indicating that PAT degradation had not started yet. (C) HPLC chromatograms of the growth medium from which Sporobolomyces cells were collected for RNA extraction after 48 h of incubation (OD595 ~ 0.8). As already known, only (Z)-ascladiol and DPA were clearly detected as the final products of PAT degradation by Sporobolomyces. (TIF 124 kb) [file 12864_2016_2550_MOESM4_ESM.tif]
